# Role of Major Endocannabinoid-Binding Receptors during Mouse Oocyte Maturation

**DOI:** 10.3390/ijms20122866

**Published:** 2019-06-12

**Authors:** Sandra Cecconi, Gianna Rossi, Sergio Oddi, Valentina Di Nisio, Mauro Maccarrone

**Affiliations:** 1 Department of Life, Health and Environmental Sciences, University of L’Aquila, 67100 L’Aquila, Italy; gianna.rossi@univaq.it (G.R.); valentina.dinisio@graduate.univaq.it (V.D.N.); 2 Faculty of Veterinary Medicine, University of Teramo, 64100 Teramo, Italy; soddi@unite.it; 3 European Center for Brain Research, Santa Lucia Foundation IRCCS, 00142 Rome, Italy; m.maccarrone@unicampus.it; 4 Department of Medicine, Campus Bio-Medico University of Rome, 00128 Rome, Italy

**Keywords:** endocannabinoids, receptors, signal transduction, meiosis, oocyte

## Abstract

Endocannabinoids are key-players of female fertility and potential biomarkers of reproductive dysfunctions. Here, we investigated localization and expression of cannabinoid receptor type-1 and -2 (CB_1_R and CB_2_R), G-protein coupled receptor 55 (GPR55), and transient receptor potential vanilloid type 1 channel (TRPV1) in mouse oocytes collected at different stages of in vivo meiotic maturation (germinal vesicle, GV; metaphase I, MI; metaphase II, MII) through qPCR, confocal imaging, and western blot. Despite the significant decrease in CB_1_R, CB_2_R, and GPR55 mRNAs occurring from GV to MII, CB_2_R and GPR55 protein contents increased during the same period. At GV, only CB_1_R was localized in oolemma, but it completely disappeared at MI. TRPV1 was always undetectable. When oocytes were in vitro matured with CB_1_R and CB_2_R but not GPR55 antagonists, a significant delay of GV breakdown occurred, sustained by elevated intraoocyte cAMP concentration. Although CBRs antagonists did not affect polar body I emission or chromosome alignment, GPR55 antagonist impaired in ~75% of oocytes the formation of normal-sized MI and MII spindles. These findings open a new avenue to interrogate oocyte pathophysiology and offer potentially new targets for the therapy of reproductive alterations.

## 1. Introduction

In mammals, the molecular processes leading to the production of female gametes are controlled by multiple interactions among different modulators, either hormones or paracrine factors [1,2]. Starting from the observation that exogenous plant-derived cannabinoids, as those present in cannabis extracts like hashish and marijuana, negatively impact fertility [3,4], several reports documented the key-role of the so called “endocannabinoid system” (ECS) on virtually all steps of female reproduction, from fertilization to oviductal transport, embryo implant and development, and pregnancy outcome [3,5,6,7,8,9]. The ECS includes: I) lipid messengers termed “endocannabinoids” (eCBs), such as anandamide (*N*-arachidonoylethanolamine, AEA) and 2-arachidonoylglycerol (2-AG); II) their receptor targets type-1 (CB_1_R) and type-2 (CB_2_R) cannabinoid receptors, G-protein coupled receptor 55 (GPR55), transient receptor potential vanilloid type 1 channel (TRPV1); III) a number of metabolic enzymes among which the eCB-cleaving fatty acid amide hydrolase (FAAH). Moreover, additional “eCB-like” compounds like *N*-palmitoylethanolamine (PEA) and *N*-oleoylethanolamine (OEA), that do not bind to CB_1_R and CB_2_R but can activate GPR55 [10], contribute to modulate eCB signalling. 

All major ECS components have been found in female and male reproductive systems [8,11,12], and eCB levels in tissues and body fluids hold promise as disease biomarkers [12,13]. Of note, the presence of a high concentration of AEA in follicular fluid is indicative of mature follicles [14,15], and significant differences of AEA and PEA in serum, and of OEA in follicular fluid, have been recorded between infertile and fertile women [16]. Furthermore, dysregulation of the ECS has been observed in polycystic ovary syndrome (PCOS) onset [17], as well as in altered human endometrial stromal cell decidualization [18].

Assessment of presence and distribution of distinct ECS components in the mammalian ovary seems necessary to clarify their roles in oocyte meiotic maturation and fertilization. In rodent follicles, distinct expression of CB_1_R, CB_2_R, and FAAH supports a different eCB-mediated response in oocyte and in follicle cells [19]. Indeed, CB_1_R is detectable in granulosa cells (GCs) of rat antral follicles, in the ovarian surface epithelium (OSE), and in luteal cells, while CB_2_R and FAAH are localized in oocytes during different stages of ovarian function, as well as in luteal cells [19]. In the human ovary, GCs has a role in the production of endocannabinoids. In fact, the AEA synthetizing enzyme *N*-acylphosphatidylethanolamine phospholipase D (NAPE-PLD) has been detected in GCs of secondary and tertiary follicles but not in oocytes [20]. Conversely, conflicting results have been obtained on CB_1_R and CB_2_R expression. Indeed, El-Talatini and colleagues found both receptors in GCs and oocytes from primordial, primary, and secondary follicles, but not in oocytes derived from antral follicles [20]. Instead, Peralta and colleagues found CB_1_R and CB_2_R in preovulatory oocytes at germinal vesicle (GV), metaphase I (MI), and metaphase II (MII) stages, with a different distribution across the germ cells depending on maturation stage [21]. In human GCs, Agirregoitia and coworkers found that CB_1_R and CB_2_R expression was differentially regulated along oocyte meiotic maturation [22]. Moreover, FAAH and the 2-AG hydrolase monoacylglycerol lipase were also detected in human germ cells during the three stages of meiotic maturation [23]. More recently, López-Cardona and collaborators linked CB_1_R signalling in bovine and mouse oocytes to the activity of two kinases involved in oocyte meiotic maturation, Akt and ERK1/2 [24,25]. Indeed, when oocytes were matured in vitro in the presence of the CB_1_R agonist HU-210, Akt phosphorylation was stimulated, while that of ERK1/2 was inhibited [24,25].

To our knowledge, there is no further information on CB_1_R and CB_2_R expression levels in mammalian oocytes, and nothing at all is known about the presence and action in these cells of the other major eCB-binding receptors GPR55 and TRPV1. Here, we sought to fill this gap by investigating the expression of CB_1_R, CB_2_R, GPR55, and TRPV1, both at mRNA and protein levels, at different stages (GV, MI, and MII) of oocyte meiotic maturation. In addition, since a role for these receptors in the process of meiotic maturation is far from clear, we performed experiments in which oocytes were matured in the presence of specific receptor antagonists to assess kinetics of meiotic maturation, polar body emission, and spindle morphology.

## 2. Results

### 2.1. mRNA Levels of eCB-Binding Receptors in GV, MI, and MII Oocytes

**Figure 1:**
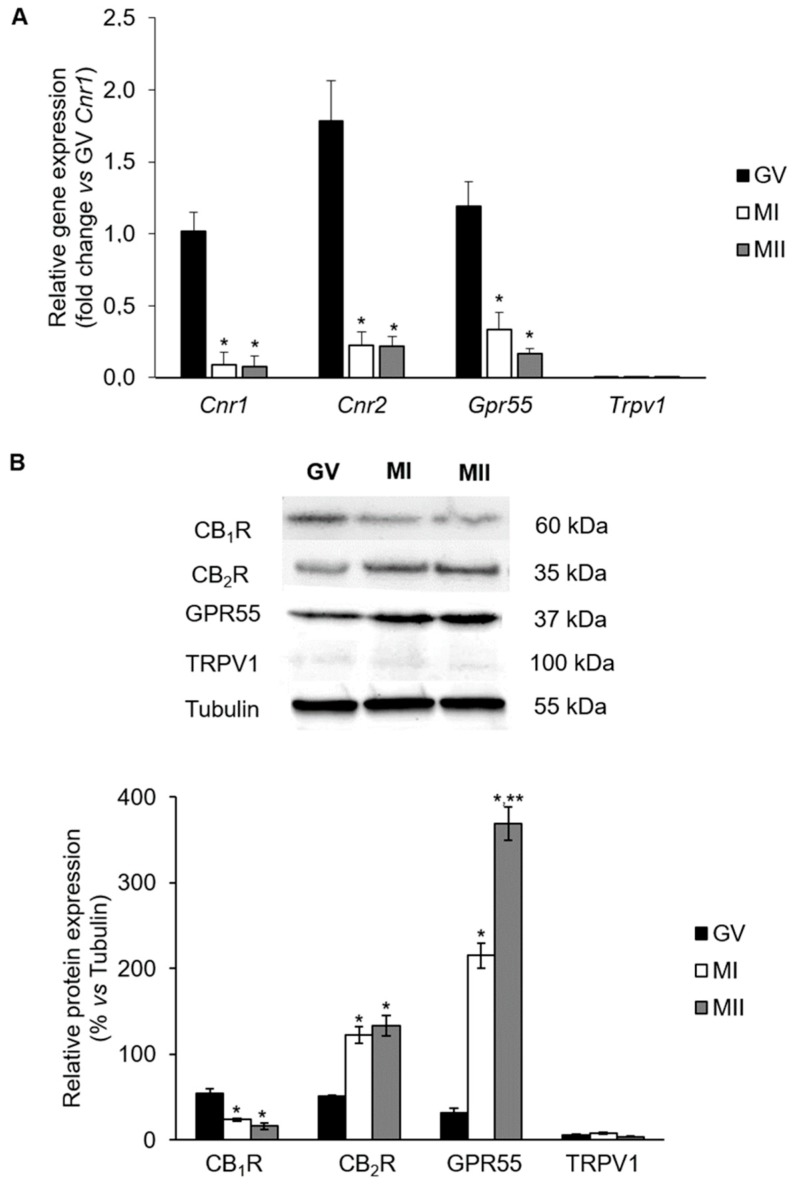
Expression levels of endocannabinoids (eCBs)-binding receptors during mouse oocyte in vivo meiotic maturation. (**A**) Real-time PCR of *Cnr1*, *Cnr2*, *Gpr55*, and *Trpv1*. Data were reported as 2^−ΔΔ*C*t^ values calculated by Delta–Delta Ct (ΔΔ*C*t) method vs. germinal vesicle (GV) (*Cnr1*) group posed equal to 1. Expression was normalized to *Actb* and values were reported as mean ± SEM of 4 independent replicates. * *p* < 0.05 vs. GV oocyte of the same experimental group. (**B**) Representative western blot and quantification of cannabinoid receptor type-1 and -2 (CB_1_R and CB_2_R), G-protein coupled receptor 55 (GPR55), and transient receptor potential vanilloid type 1 channel (TRPV1) protein contents. Data are expressed as mean ± SEM of each receptor content after normalization with α/β-tubulin, used as loading control. Experiments were repeated 3 times. * *p* < 0.05 vs. GV oocyte of the same experimental group; ** *p* < 0.05 vs. MI of the same experimental group. GV = germinal vesicle; MI = metaphase I; MII = metaphase II.

Data obtained from real-time PCR analysis showed that mRNA levels of *Cnr1*, *Cnr2*, and *Gpr55* (the genes encoding for CB_1_R, CB_2_R, and GPR55, respectively) decreased dramatically during in vivo meiotic maturation, from GV to MI and MII stage (Figure 1A; vs. GV, *p* < 0.05). Conversely, *Trpv1* (the gene encoding for TRPV1) mRNA expression was very low throughout meiotic maturation, with no significant differences between GV, MI, and MII (Figure 1A; *p* > 0.05).

### 2.2. Protein Levels of eCB-Binding Receptors in GV, MI, and MII Oocytes

Receptors of eCBs were immunodetected in lysates of in vivo matured oocytes. At GV stage, similar levels of CB_1_R, CB_2_R, and GPR55 proteins were found (Figure 1B; *p* > 0.05). At MI, CB_1_R content was significantly reduced (Figure 1B; GV vs. MI and MII, *p* < 0.05), while CB_2_R and even more GPR55 expression levels showed a sharp increase (Figure 1B; GV vs. MI: *p* < 0.05). As reported in Figure 1B, in MII oocytes only GPR55 content raised dramatically (Figure 1B; GPR55: GV vs. MI vs. MII, *p* < 0.05; CBR2: MI vs. MII, *p* > 0.05). TRPV1 signal was always barely detectable at any meiotic stage analyzed (Figure 1A,B; *p* > 0.05).

### 2.3. Localization of eCB-Binding Receptors in GV, MI, and MII Oocytes

**Figure 2:**
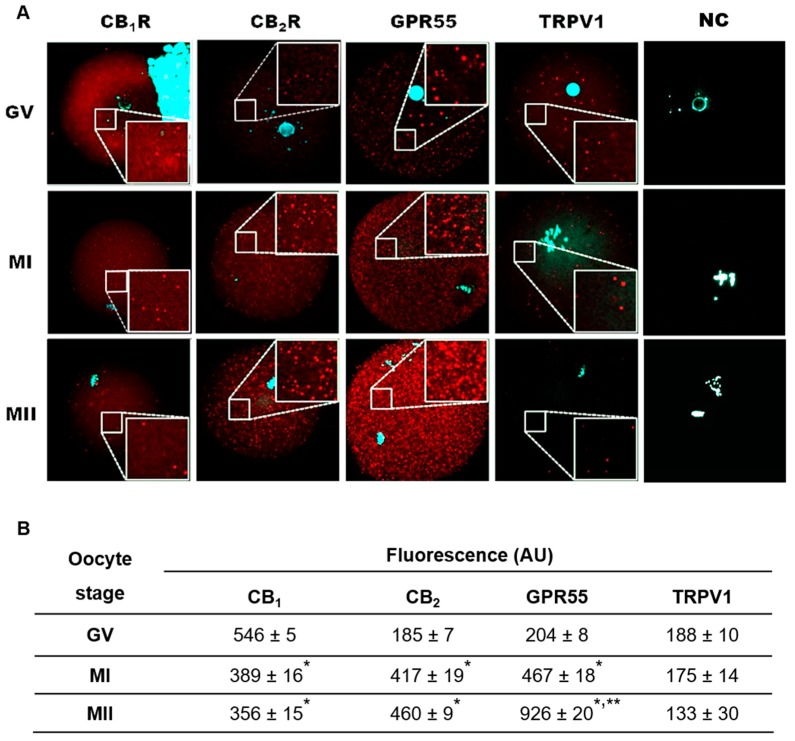
(**A**) Localization of CB_1_R, CB_2_R, GPR55, and TRPV1 receptors in mouse oocytes collected at various stages of in vivo meiotic maturation. Receptors were labelled with Cy-3 (red), DNA was counterstained by DAPI (cyan). In the upper right-hand corner of GV CB_1_R, the strong DAPI staining is due to undetached cumulus cell nuclei. Each image was taken at the equatorial plan of the oocyte. Magnification: ×630. Each inset represents a magnified part of ooplasm. GV = germinal vesicle; MI = metaphase I; MII = metaphase II; NC = negative control. (**B**) Mean fluorescence of CB_1_R, CB_2_R, GPR55, and TRPV1 receptors in mouse oocytes collected at different stages of meiotic maturation. Values are expressed as arbitrary units (AU) and are reported as mean ± SEM of 6 oocytes from 3 independent experiments. * *p* < 0.05 vs. GV oocyte of the same experimental group; ** *p* < 0.05 vs. MI of the same experimental group.

In these experiments, the immunolocalization of CB_1_R, CB_2_R, GPR55, and TRPV1 was carried out in oocytes collected in vivo at GV, MI, and MII. At GV stage, all receptors showed a homogeneous distribution over the entire cytoplasm even if CB_1_R immunostaining was more intense than that of CB_2_R and GPR55 (Figure 2A,B). In MI oocytes, while CB_1_R signal intensity was restricted to some cytoplasmic dots, that of CB_2_R and GPR55 increased, being still homogeneously distributed across the germ cell (Figure 2A,B). At MII, fluorescence was very faint for CB_1_R, unchanged for CB_2_R but not for GPR55, as it was remarkably enhanced (Figure 2A,B). In keeping with molecular data, signal intensity of TRPV1 was barely detectable throughout meiotic maturation (Figure 2A,B).

**Figure 3:**
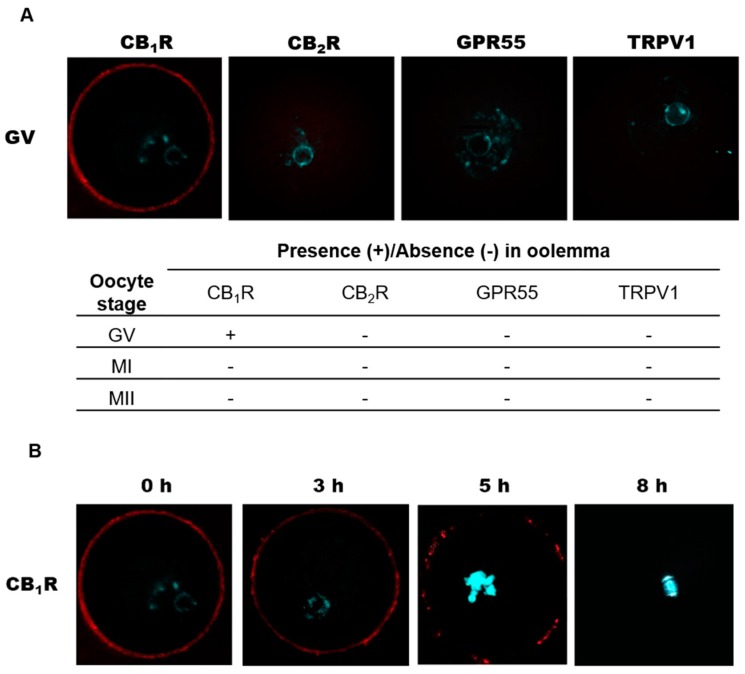
Distribution of CB_1_R, CB_2_R, GPR55, and TRPV1 in the oolemma and CB_1_R localization at oolemma during in vivo GV–MI transition. (**A**) Only GV oocytes showed detectable signals. Qualitative data are expressed as presence (+) or absence (-) of fluorescence in 15 oocytes/sample. (**B**) CB_1_R distribution, analyzed at different times after human chorionic gonadotropin (hCG), changed from uniformly homogeneous (0–3 h) to dotted clusters (5 h), until complete disappearance at MI (8 h). Each image was taken at the equatorial plan of the oocyte. Cy-3: red, DAPI: cyan. Magnification: ×630.

Analysis of CB_1_R localization at oocyte plasma membrane (oolemma) revealed its presence at GV stage, while it failed to detect it at MI and MII stage (Figure 3A). Unlike CB_1_R, the other 3 receptors were never found at oolemma at any meiotic stage analyzed (GV: Figure 3A; MI, MII: data not shown). To further characterize CB_1_R dynamics during the transition from GV to MI, oocytes were collected at 0, 3, 5, and 8 h after human chorionic gonadotropin (hCG). It was found that the homogenous distribution of CB_1_R recorded at 0 h was lost 3 h later, when receptor localization was restricted to small microdomains, probably associated with lipid rafts (Figure 3B). At 5 h, CB_1_R was almost completely compartmentalized in few microdomains, and its signal disappeared from oolemma when oocytes reached the MI stage (Figure 3B).

### 2.4. Effects of Receptor Antagonists on Intraoocyte cAMP Concentration

**Figure 4:**
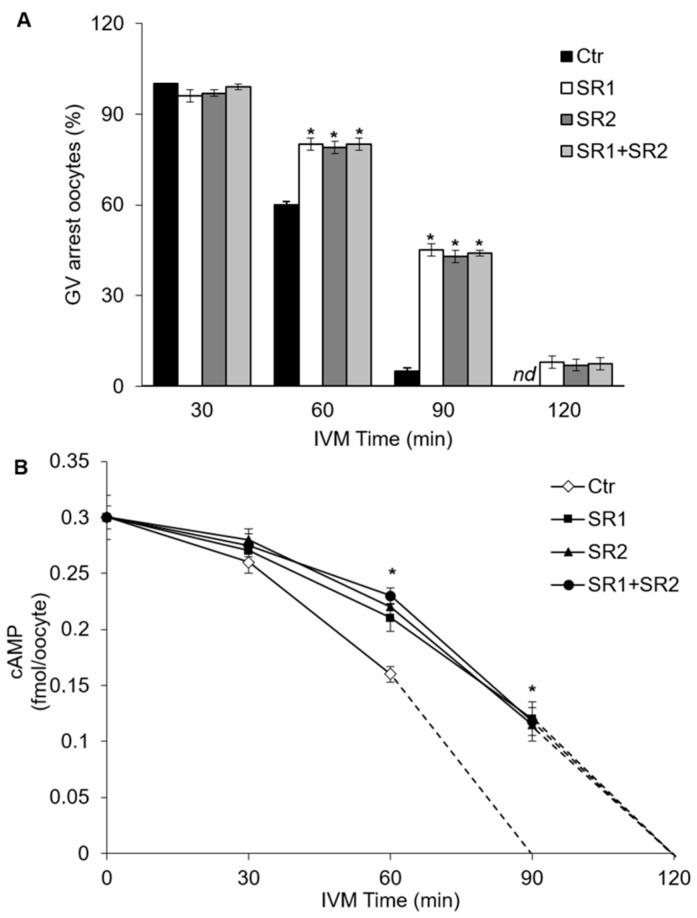
Effects of CBRs antagonists on the kinetics of oocyte germinal vesicle breakdown (GVBD) and on cAMP concentration. (**A**) Oocyte–cumulus cell complexes (OCCs) were matured in vitro in the presence of 0.05% DMSO (Ctr) or SR1, SR2, and SR1 + SR2 (0.5 μM) for 30, 60, 90, and 120 min. Data are expressed as percentage of GV arrested/total oocytes. *nd*: not detectable (**B**) cAMP concentration (fmol/oocyte) was assayed in oocytes cultured in vitro for 30, 60, 90, and 120 min as Ctr or in the presence of SR1, SR2, and SR1 + SR2 (0.5 μM). Data are expressed as mean ± SEM of 4 independent experiments. * *p* < 0.05 vs. Ctr oocytes of the same experimental group. *nd*: values below the limit of detection of the assay (0.1 pmol/mL).

In these experiments, the effects of SR1 and SR2, antagonists of CB_1_R and CB_2_R respectively, on the kinetics of meiotic resumption and cAMP concentration were tested. Antagonists were used alone or in combination at the final concentration of 0.5 µM. As shown in Figure 4A, 30 min after starting culture, almost all oocytes were still arrested at GV stage (Ctr vs. SR1, SR2, SR1+SR2; *p* > 0.05). At 60 min, germinal vesicle breakdown (GVBD) occurred in about 40% of Ctr oocytes, and in about 20% of oocytes treated with SR1, SR2 or both antagonists (Figure 4A; Ctr vs. SR1, SR2, SR1+SR2; *p* < 0.05). After 90 min, about 95% of Ctr oocytes underwent GVBD as compared with about 56% of oocytes treated with SR1, SR2 or both antagonists (Figure 4A; Ctr vs. SR1, SR2, SR1+SR2; *p* < 0.05). At a later time (120 min), almost all oocytes had resumed meiosis (Ctr vs. SR1, SR2, SR1+SR2; *p* > 0.05). When both antagonists were present, the percentages of oocytes resuming meiosis were similar to those of SR1 or SR2 at any time point tested (Figure 4A; SR1+SR2 vs. Ctr; *p* > 0.05).

On the basis of the kinetics of meiotic resumption, in the next set of experiments we tested the hypothesis that CB_1_R and CB_2_R, both able to activate G_αi_ proteins [26], could be involved in meiotic resumption by modulating cAMP intraoocyte concentration [27,28]. To this end, cAMP concentration was determined in oocytes cultured in vitro up to 120 min either in the absence (Ctr) or in the presence of SR1, SR2 or SR1+SR2. At the beginning of culture, cAMP content was 0.30 ± 0.01 fmol/oocyte, and 30 min later it showed a slight yet not significant decrease under all experimental conditions (Figure 4B; *p* > 0.05). A sharp decrease in cAMP concentration occurred at 60 min in SR1-, SR2-, and SR1+SR2-treated cells (~0.21 ± 0.012 fmol/oocyte) and even more in Ctr (0.16 ± 0.007 fmol/oocyte) (Figure 4B, vs. Ctr; *p* < 0.05). At 90 min, cAMP concentration was undetectable in Ctr oocytes, while it was ~0.11 ± 0.015 fmol/oocyte in SR1-, SR2-, and SR1+SR2-treated cells (Figure 4B; Ctr vs. SR1, SR2, SR1+SR2, *p* < 0.05). After 120 min, cAMP was no longer detectable in all groups (Figure 4B; Ctr vs. SR1, SR2, SR1+SR2, *p* > 0.05). Similar results were obtained in the presence of both antagonists (Figure 4B).

### 2.5. Effects of Receptor Antagonists on Polar Body I Emission and Spindle Morphology

In these experiments, it was ascertained whether antagonists of CB_1_R, CB_2_R, and GPR55 could affect polar body I (PBI) emission and/or the morphology of MI-MII spindles. The presence of SR1, SR2 or ML193 (0.5 µM) did not perturb in vitro maturation (IVM), as the percentage of oocytes reaching MI (>90%; vs. Ctr, *p* > 0.05) and MII stage (~80% PBI; vs. Ctr, *p* > 0.05) were comparable with control. Similar results were obtained when oocytes underwent IVM in the presence of the 3 antagonists (MI: >90%; vs. Ctr, *p* > 0.05; MII: ~82% PBI; vs. Ctr, *p* > 0.05) (Table 1).

**Figure 5:**
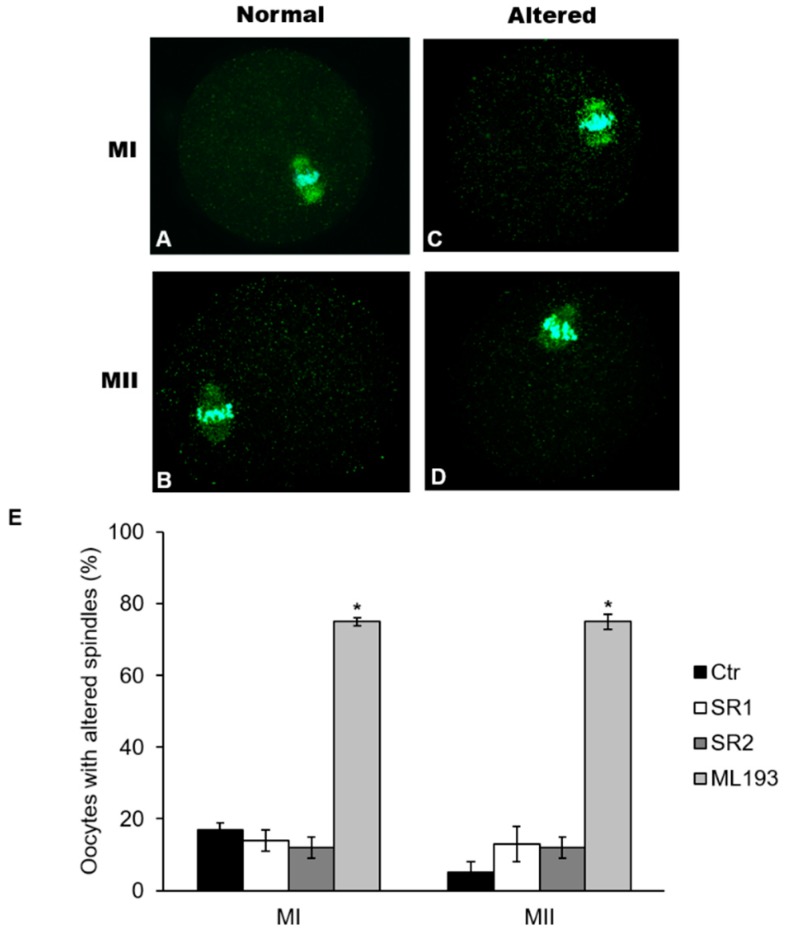
MI and MII spindle morphology and size. To obtain MI oocytes, OCCs were cultured in presence or absence (Ctr) of SR1, SR2, and ML193 (0.5 μM) for 8 h. To obtain MII oocytes, OCCs were collected 8 h after hCG and cultured in vitro for 5 h. Spindles were stained with anti-α/β-tubulin antibody (green) and chromosomes with DAPI (cyan). Representative images of normal MI (**A**) and MII (**B**) spindles and of altered MI (**C**) and MII (**D**) spindles. Magnification: ×630. (**E**) Oocytes percentages of MI and MII altered spindles in presence of CBRs antagonists. Data are expressed as mean ± SEM of 3 independent experiments. * *p* < 0.05 vs. Ctr oocytes of the same experimental group.

The presence of SR1 or SR2 did not affect normal chromosome alignment at both metaphase plates (>95%; vs. Ctr, *p* > 0.05) nor spindle morphology, as more than 80% of oocytes had a mean spindle length (MI: ~35.25 ± 0.19 µm; MII: ~32.39 ± 0.24 µm) and area (MI: ~569.78 ± 3.17 µm^2^; MII: ~481.52 ± 1.64 µm^2^) comparable with Ctr (Figure 5A,B; *p* > 0.05). Conversely, ML193 dramatically affected overall spindle morphology, but not chromosome alignment, at either MI or MII stage. Indeed, about 75% of MI and MII oocytes cultured in the presence of this GPR55 antagonist displayed spindles significantly shorter (MI: 24.34 ± 0.44 µm; MII: 22.96 ± 0.30 µm) and with smaller areas (MI: 376.44 ± 2.42 µm^2^; MII: 247.60 ± 1.31 µm^2^), as compared with Ctr (Figure 5C–E; *p* < 0.05). 

## 3. Discussion

Our results demonstrate that (i) the four major eCB-binding receptors are expressed in mouse oocytes, but CB_1_R, CB_2_R, and GPR55 expression changes throughout in vivo meiotic maturation. Conversely, TRPV1 expression is always low/undetectable; (ii) CB_1_R and CB_2_R can play a role in meiotic resumption, while GPR55 could be involved in spindle organization. 

We found that *Cnr1*, *Cnr2*, and *Gpr55* mRNAs are expressed in oocytes at GV, while their amounts decrease drastically at MI and MII in keeping with the well-known repression of transcription that follows GVBD [29]. Part of these data are different from those recently reported on CB_2_R mRNA, that was found to be expressed throughout in vivo maturation [25]. This could be due to the different methodology used for MI and MII oocyte recruitment utilized in the two studies. Indeed, we obtained MI by puncturing ovarian follicles 8 h after hCG and MII oocytes from the oviducts 12 h after hCG, while López-Cardona and colleagues retrieved both MI and MII from the oviducts 14 h after hCG [25]. As for humans, only CB_1_R transcripts were reported in mouse oocytes [21], though cells of different meiotic stages were pooled together, thus preventing any conclusion on their modulation during meiosis.

In our experiments, localization of CB_1_R at GV oolemma is modified during GVBD, as CB_1_R is progressively clustered in large microdomains probably associated with lipid-rafts, a preferential localization documented also in neuronal cells [30]. We hypothesized that CB_1_R disappearance from oolemma could be relevant for fertilization and/or early embryo development. It has been demonstrated that in the ampulla, where fertilization occurs, AEA concentration is low, but that spermatozoa here present have undergone CB_1_R-dependent acrosome reaction (AR) in the isthmus, where AEA concentration is high [12,31]. As a consequence, we cannot exclude that the permanence of CB_1_R at the oocyte plasma membrane could trigger inappropriate signalling during the meiotic maturation and fertilization process. Moreover, it is worth to notice that both CB_1_R and CB_2_R are expressed in the early embryo (CB_2_R in the zygote and CB_1_R from 2 cell-embryo stage onward [32]), but with different and still unexplained roles. In fact, while CB_2_R is unresponsive to agonist stimulation and its role has not been yet identified [25,33], CB_1_R plays a key role in embryonic development [33,34]. To date, nothing is yet known about the other receptors. 

Our results show that CB_2_R, GPR55, and TRPV1 receptors have different dynamics during meiotic maturation. While TRPV1 is weakly expressed at any meiotic stage, CB_2_R content increases from GV to MI and that of GPR55 throughout the whole meiotic maturation. Our results on CB_1_R and CB_2_R are different from those reported by López-Cardona and colleagues [25], who showed that localization of CB_1_R is differentially influenced by in vivo (periphery of the oocyte from GV to MII) or in vitro maturation (firstly in the cytoplasm at GV, and then peripherally at MII). Such a different distribution is unexpected, since oocytes collected at GV display identical properties regardless of conditions adopted for subsequent (in vivo or in vitro) maturation. Also, for CB_2_R, López-Cardona and colleagues [25] found a homogeneous immunofluorescence in the cytoplasm at all meiotic stages, while we recorded a sharp increase of CB_2_R signal from GV to MI. Although the different procedures used to collect oocytes might explain this discrepancy, the quantification of protein expression performed here strongly supports confocal images.

An additional point of interest is that López-Cardona and colleagues [25] concluded that CB_1_R is more important than CB_2_R in the control of oocyte maturation. Instead, here a role for both receptors in the control of meiotic resumption is supported by experiments with selective receptor antagonists SR1 and SR2. Indeed, the significant delay of GVBD and higher cAMP concentration allow us to conclude that both receptors can modulate oocyte adenylyl cyclase activity, possibly through G_αi_ proteins coupled to them, as shown in other cell systems [26]. It is well known that meiotic arrest in GV oocytes is maintained by a high intra-oocyte cAMP concentration [27,28]. The autonomous production of this cyclic nucleotide is ensured by the presence of active GPR3 receptors coupled to G_αs_ proteins, which maintains the high levels of cAMP necessary for GV arrest via activation of adenylyl cyclase type 3 [28,35]. Following gonadotropin stimulation, cAMP concentration in the oocyte falls down sharply around the time of GVBD, due to gap junction closure and PDE3A activation [35,36,37]. Lowther et al. [38] found that also GPR3 endocytosis through a beta-arrestin/GRK-independent mechanism participates in meiotic resumption. Since we observed that CB_1_R becomes entrapped in large clusters soon after GVBD and disappears from the oolemma at MI, we hypothesize that the concomitant internalization of CB_1_R and GPR3 could be part of the mechanism(s) involved in the control of meiotic resumption. As a matter of fact, receptor endocytosis modulates in the control of GVBD in vertebrate oocytes [39]. The co-existence in GV oocytes of serpentine receptors coupled to G_αi_ proteins, localized either in plasma membranes (CB_1_R) [26,30] or intracellularly (CB_2_R) [40], could be instrumental to properly regulate intra-oocyte cAMP concentration and GVBD [41]. This hypothesis is further corroborated by the presence of functional microdomains for cAMP production in plasma membrane and cytoplasm of many cell types [42], as in fish oocytes [43] and in rat oocyte nucleus [44].

From our experiments it is also evident that the use of SR1 and SR2 during IVM does not induce any variation in meiotic spindle structure and in the percentage of MII oocytes extruding normal PBI. This result is in agreement with the observation by López-Cardona and colleagues [25] on *Cnr1* or *Cnr2* knockout mice, although the presence of both receptors during oocyte growth and maturation is essential for successful fertilization and embryogenesis. 

Conversely, a novel role of GPR55 in the formation of MI and MII spindles has been revealed by experiments with its antagonist. In fact, despite nearly all the oocytes cultured with ML193 extrude normal PBI, a high percentage of them exhibits variation in spindle size at both MI (75%) or MII (75%) stage compared with Ctr (19% for MI, 5% for MII), without compromising chromosome alignment at both metaphase plates. To the best of our knowledge, this is the first time that a link between GPR55 and spindle organization has been found in mammalian cells. In mammalian oocytes, spindle length is controlled by a complex interplay of many proteins [45,46,47], and its normal length is considered a marker of oocyte quality [48]. Recently, Wang and collaborators proposed that spindle size and timing of meiotic progression are efficiently controlled more by cytoplasmic than by nuclear components [49]. In keeping with this hypothesis, dynein, dynactin and NuMA are able to control microtubule length, while γ-tubulin regulates the polymerization of α-tubulin [50]. Although we do not know how GPR55 could affect spindle morphology yet, here we show that GPR55 protein is entirely localized in the cytoplasm and its expression raises in a stepwise manner from GV to MI and, more greatly, to MII. This finding supports a possible role for this receptor not only in meiotic maturation but, at later times, also in fertilization and embryo development. It is of interest that GPR55 can mediate Ca^2+^ mobilization from IP3-sensitive intracellular stores via G_q_, G_α12_, RhoA, PLC, and actin [51], and that an essential role for GPR55 activation in the Ca^2+^-dependent regulation of human sperm motility and capacitation has been proposed [52,53]. How GPR55 can participate in the oocyte spindle formation is currently under study in our laboratories. Altogether, these observations suggest that we are still far from understanding the importance of the whole ECS in female reproduction.

## 4. Materials and Methods

### 4.1. Chemicals

Hepes-buffered Eagle’s minimal essential medium (MEM-HEPES) and MEM-ALPHA modification (αMEM), penicillin, and streptomycin were purchased from ThermoFisher Scientific (MA, USA). Pregnant mare serum gonadotropin (PMSG, Folligon) and human chorionic gonadotropin (hCG, Corulon) were obtained from Intervet International B. V. (AN Boxmeer, Nederland). SR141716A (SR1; cat. 0923/10) and SR144528 (SR2; cat. 5039/10) were obtained from Tocris Bioscience, Bristol, UK). ML193 trifluoroacetate (ML193, cat. SML1340) was obtained from Sigma-Aldrich (St. Louis, MO, USA). Primary antibodies against rabbit CB_1_R (cat. 101500), CB_2_R (cat. 101550), GPR55 (cat. 10224), and specific blocking peptides for CB_1_R (cat. 301500) and CB_2_R (cat. 301550) and GPR55 (cat. 10225) antibodies were obtained from Cayman Chemical (Anne Arbore, MI, USA). Rabbit TRPV1 (cat. TA336871) antibody was obtained from OriGene Technologies, Inc. (Rockville, MD, USA). Cyanine 3 bisacid (Cy-3) anti-rabbit (cat. A10520) and Alexa Fluor 488 anti-mouse (cat. A32723) used as secondary antibodies for immunofluorescence analysis, and goat anti-rabbit horseradish peroxidase (HRP, cat. 111-035-003), used as secondary antibody for western blotting, were obtained from ThermoFisher Scientific. All the other reagents were purchased from Sigma-Aldrich (St. Louis, MO, USA), and were of the purest analytical grade.

### 4.2. Animals

*Mus musculus* Swiss CD1 female mice (23–25 day old; Charles River Laboratories, Lecco, Italy) were housed in an animal facility under controlled temperature (21 ± 1 °C) and light (12 h light/day) conditions, with free access to food and water. 

All mice were injected with PMSG (5 IU, i.p.). Forty-two to forty-four hours later, mice were sacrificed to obtain preovulatory germinal vesicle (GV)-stage oocytes or were injected with hCG (5 IU, i.p.) and sacrificed 3–12 h after hCG injection (depending on experimental protocols). MI and MII oocytes were retrieved 8 and 12 h after hCG injection, respectively.

### 4.3. Ethical Approval

All experimental procedures involving animals and their care were performed in conformity with national and international laws and policies (European Economic Community Council Directive 86/609, OJ 358, 1, 12 December, 1987; European Parliament Council Directive 2010/63/EU, OJ L 276, 20 October 2010; Italian Legislative Decree 116/92, Gazzetta Ufficiale della Repubblica Italiana n. 40, 18, February 1992; National Institutes of Health Guide for the Care and Use of Laboratory Animals, NIH publication no. 85-23, 1985). The project was approved by the Italian Ministry of Health and the internal Committee of the University of L’Aquila. The method of euthanasia consisted of an inhalant overdose of carbon dioxide (CO_2_, 10–30%), followed by cervical dislocation. All efforts were made to minimize suffering. A total number of 200 mice were utilized to perform all the experimental procedures.

### 4.4. Collection of In Vivo Matured Oocytes

Fully-grown, GV-stage oocytes surrounded by cumulus cells (oocyte-cumulus cell complexes, OCC) were collected in MEM-HEPES supplemented with 0.23 mM pyruvic acid, 2 mM l-glutamine and 0.3% BSA (here referred as MEM), and immediately devoid of cumulus cells by gentle pipetting. MI and MII oocytes were recovered from ovaries 8 h after hCG and from fallopian tubes 12 h after hCG, respectively. When needed, cumulus cells were removed by a brief hyaluronidase treatment [54]. Oocytes were immediately used for morphological or molecular analysis. 

### 4.5. Quantitative Real-Time PCR Analysis

Total RNA was extracted from GV, MI and MII oocytes (20 oocytes/sample) using the RNeasy extraction kit (Qiagen, Crawley, UK) as suggested by the manufacturer. Starting with 100 ng of RNA, complementary DNA (cDNA) was prepared using M–MLV reverse transcriptase kit (ThermoFisher Scientific, Waltham, MA, USA). Quantitative PCR analysis was performed using SYBR Green I Master and the LightCycler 480 System (Roche, Basel, Switzerland) on a DNA Engine Opticon 2 Continuous Fluorescence Detection System (BioRad, Hercules, CA, USA). The reaction was performed using the following qRT-PCR program: 95 °C for 10 min, followed by 40 amplification cycles of 95 °C for 10 s, 57 °C for 30 s, and 72 °C for 30 s. The primer used for the amplification of *Cnr1*, *Cnr2*, *Gpr55*, and *Trpv1* [55,56] were listed in Table 2 and all the data were normalized to the endogenous reference gene β-Actin. Relative quantitation of mRNAs was performed by the comparative ΔΔ*C*t method [57].

### 4.6. Western Blotting Analysis

GV, MI, and MII oocytes (150 oocytes/sample) were lysed in sample buffer containing protease inhibitors (2 mM phenylmethyl sulphonyl fluoride, 10 µg/mL aprotinin, 0.1 mM sodium pyrophosphate, 10 mM sodium fluoride, and 1 mM sodium orthovanadate). Lysates were separated by electrophoresis and transferred to nitrocellulose membranes (Hybond C Extra, Amersham, UK). Membranes were incubated with antibodies against CB_1_R (1:200), CB_2_R (1:200), GPR55 (1:200), and TRPV1 (1:200) overnight at 4 °C. HRP-conjugated goat anti-rabbit IgG (1:5000) was used as secondary antibody (1 h, room temperature); peroxidase activity was detected using a SuperSignal West Pico Chemiluminescent substrate. Membranes were examined by Alliance LD2-77WL imaging system (Uvitec, Cambridge, UK). Densitometric quantification was performed with the public-domain software NIH Image 167 V.1.62 and standardized using tubulin as loading control. Negative controls were prepared using specific blocking peptide (for CB_1_R and CB_2_R and GPR55). For the anti-TRPV1 antibody used in this study, there are no blocking peptides commercially available.

### 4.7. Immunofluorescence

To detect presence and distribution of receptors at GV, MI and MII stage, oocytes (15/sample) were fixed in 4% paraformaldehyde for 10 min at r.t., permeabilized with 0.1% Triton X-100 for 30 min at 37 °C [54,58]. Afterwards, oocytes were incubated with the following primary antibodies diluted in a PBS blocking solution (containing 2% BSA, 2% powder milk, 2% normal goat serum, 0.1 M Glycine, and 0.01% Triton X-100): CB_1_R (1:100), CB_2_R (1:100), GPR55 (1:200), and TRPV1 (1:200) for 1 h at 37 °C. To detect receptor presence at oocyte plasma membrane (oolemma), oocytes were firstly incubated in Tyrode’s solution at pH 2.5 to remove zona pellucida (zona-free, ZF), and then fixed in 4% paraformaldehyde (0.2% BSA in 0.1 M PBS pH 7.4). Afterwards, oocytes were incubated for 1 h at 37 °C with the following primary antibodies diluted in 0.2% BSA-PBS: CB_1_R (1:100), CB_2_R (1:100), GPR55 (1:200), and TRPV1 (1:200). Oocytes were then incubated with Cy-3 anti-rabbit secondary antibody (1:200) for 1 h at 37 °C, mounted using 1.5 µl of 50% glycerol/PBS solution containing sodium azide and DAPI (1:1000) to label nuclei [59]. To monitor the distribution of CB_1_R in the oolemma during the GV–MI transition, ZF-oocytes were collected at different times after hCG injection (0, 3, 5, 8 h; 15 oocytes/time point), and incubated with CB_1_R antibody (1:100)-Cy-3 anti-rabbit secondary antibody (1:200) for 1 h at 37 °C each. 

For each set of experiments, negative controls (NC) were prepared using specific blocking peptide (for CB_1_R and CB_2_R and GPR55) and omitting the primary antibody for TRPV1 before addition of the secondary antibody. For the anti-TRPV1 antibody used in this study there are no blocking peptides commercially available. All the oocytes were observed by confocal microscopy (Leica System TCS SP5 confocal microscope, Wetzlar, Germany). Images were taken at the equatorial plan using the LAS AF software (Leica Microsystems). 

For image analysis, data from high-resolution images of 6 oocytes from 3 independent experiments were acquired for each sample. Quantification of the intracellular mean fluorescence of CBRs was carried out using the public-domain software NIH Image 167 V.1.62 after the subtraction of the background intensity calculated from the images of NC.

### 4.8. Effects of CB_1_R and CB_2_R Antagonists on Intraoocyte cAMP Content

OCCs were collected in MEM supplemented with cilostamide (1 µM) to maintain meiotic arrest [60]. After washing, OCCs were (i) in part devoid of somatic cells to obtain GV stage oocytes (*t* = 0) that were immediately stored at −80 °C; (ii) in part cultured at 37 °C in 5% CO_2_ for 30, 60, 90, and 120 min in the absence (Ctr) or presence of CB_1_R antagonist SR141716 (SR1) and CB_2_R antagonist SR244528 (SR2), alone or in combination. The antagonists were all used at 0.5 µM because it was the lowest effective dose after preliminary experiments, and for CB_1_R and CB_2_R was in line with previous study [57]. Culture medium was alpha MEM supplemented with 0.23 mM pyruvate, 2 mM l-glutamine and 0.05% DMSO (hereafter referred as αMEM-DMSO). At each time point, OCCs were deprived of cumulus cells to record the percentage of GVs, according to the presence or absence of the GV in the ooplasm. 

The amount of cAMP was determined in groups of 120 oocytes incubated stored after GV assessment, by using a Cyclic AMP EIA Kit (581001, Cayman Chemical Company, Anne Arbore, MI, USA) according to manufacturer’s instructions. Absorbance at 420 nm was measured in a Model 550 microplate reader (BioRad).

### 4.9. Effects of Receptor Antagonists on Polar Body I Emission and Spindle Formation

To evaluate the effects of antagonists on the morphology of MI spindle, OCCs were cultured for 8 h in 300 μL αMEM-DMSO in the absence (Ctr, *n* = 30 oocytes) or presence of 0.5 µM SR1 (*n* = 45 oocytes), SR2 (*n* = 45 oocytes), ML193 (*n* = 80 oocytes), or a combination of the three antagonists (SR1 + SR2 + ML193; *n* = 50 oocytes). Following cumulus cells removal, only oocytes undergoing GVBD were fixed as described in the following procedure. 

The analysis of antagonists’ effect on PBI and MII spindles were performed by retrieving OCCs 8h after hCG, i.e., at MI in vivo, and then by culturing them in the absence (Ctr, *n* = 30 oocytes) or presence of 0.5 µM SR1 (*n* = 30 oocytes), SR2 (*n* = 50 oocytes) or ML193 (*n* = 80 oocytes), or a combination of the three antagonists (SR1+SR2+ML193; *n* = 50 oocytes) for 5 h. This experimental design was chosen in order to reduce the times of oocyte in vitro culture. By the end of culture period, the percentage of normal PBI was recorded, and oocytes were then fixed as described above. Afterwards, oocytes were incubated for 1 h at 37 °C with anti-tubulin primary antibody (1:100) and then with anti-mouse secondary antibody conjugated with Alexa Fluor 488 (1:1000). Chromosomes were labelled with DAPI (1:1000) [58,59]. Spindle sizes (length [49] and area [61]) were measured by the software ZEN 2009 Light Edition (Carl Zeiss MicroImaging GmbH), as previously described [49]. 

### 4.10. Statistical Analyses

All experiments were performed at least 3 times, and data obtained were expressed as the mean ± S.E.M. Statistical analysis was performed using ANOVA followed by the Tukey-Kramer post-test for comparison of multiple groups, by Bonferroni post-test for comparison among treatments and control groups and by the chi-square test for comparison of percentages. Values of *p* < 0.05 were considered significantly different.

## 5. Conclusions

Our results demonstrate that in mouse oocytes the major eCB-binding receptors are differentially expressed and modulated during meiotic maturation. Present data support a prominent role for CB_1_R and CB_2_R in the control of meiosis resumption, and the engagement of GPR55 in MI and MII spindle organization. These findings open a new avenue to interrogate oocyte pathophysiology and offer potentially novel biomarkers for fertility problems.

## Figures and Tables

**Table 1:** Percentages of oocytes at MI or MII stage after in vitro maturation (IVM) in the presence or absence (Ctr) of CBRs antagonists.

Condition	Num. of Oocytes	MI (%)	MII (%)
Ctr	30	96.02 ± 3.98	81.25 ± 2.13
SR1	45	95.80 ± 3.15	82.93 ± 1.42
SR2	45	94.56 ± 5.03	80.95 ± 3.26
ML193	80	95.51 ± 4.36	81.16 ± 2.70
SR1+SR2+ML193	50	94.83 ± 2.91	81.60 ± 2.24

Note: For complete and detailed information on the different IVM procedures see Section 4.9 Materials and Methods. All oocytes were analyzed by confocal microscopy for the evaluation of spindle morphology.

**Table 2:** List of the primers used for quantitative real-time PCR analysis.

Gene	Corresponding Protein	PCR Primers	Annealing T (°C)	Reference
*Cnr1*	CB_1_R	Fw: 5′-CCAAGAAAAGATGACGGCAG-3′ Rev: 5′-AGGATGACACATAGCACCAG-3′	57	[55]
*Cnr2*	CB_2_R	Fw: 5′-TCGCTTACATCCTTCAGACAG-3′ Rev: 5′-TCTTCCCTCCCAACTCCTTC-3′	57	[55]
*Gpr55*	GPR55	Fw: 5′-ATTCGATTCCGTGGATAAGC-3′ Rev: 5′-ATGCTGATGAAGTAGAGGC-3′	57	[56]
*Trpv1*	TRPV1	Fw: 5′-TGAACTGGACTACCTGGAAC-3′ Rev: 5′-TCCTTGAAGACCTCAGCATC-3′	57	[56]
*Actb*	Actin	Fw: 5′-CTGTCGAGTCGCGTCCACCC-3′ Rev: 5′-GCTTTGCACATGCCGGAGCC-3′	57	[55]

## Abbreviations

2-AG	2-arachidonoylglycerol
AEA	*N*-arachidonoylethanolamine
AU	Arbitrary units
BSA	Bovine Serum Albumin
cAMP	Cyclic adenosine monophosphate
CB_1_R	Cannabinoid receptor type-1
CB_2_R	Cannabinoid receptor type-2
CTR	Control
Cy-3	Cyanine 3 bisacid
DAPI	4′,6-diamidino-2-phenylindole
DMSO	Dimethyl sulfoxide
eCBs	Endocannabinoids
ECS	Endocannabinoid system
ERK 1/2	Extracellular signal-regulated kinases 1/2
FAAH	Fatty acid amide hydrolase
GCs	Granulosa cells
GPR3	G protein-coupled receptor 3
GPR55	G-protein coupled receptor 55
GRK	G protein-coupled receptor kinase
GV	Germinal vesicle
GVBD	Germinal vesicle breakdown
hCG	Human chorionic gonadotropin
HU-210	1,1-Dimethylheptyl-11-hydroxy-tetrahydrocannabinol
IP3	Inositol trisphosphate
IVM	In vitro maturation
MI	Metaphase I
MII	Metaphase II
ML193	*N*-[4-[[(3,4-dimethyl-5-isoxazolyl)amino]sulfonyl]phenyl]-6,8-dimethyl-2-(2-pyridinyl)-4-quinolinecarboxamide
NAPE-PLD	*N*-acyl phosphatidylethanolamine-specific phospholipase D
NC	Negative control
NuMA	Nuclear mitotic apparatus protein 1
OCCs	Oocyte-cumulus complexes
OEA	*N*-oleoylethanolamine
OSE	Ovarian surface epithelium
PBI	Polar body I
PCOS	Polycystic ovary syndrome
PDE3A	Phosphodiesterase 3A
PEA	*N*-palmitoylethanolamine
PLC	Phospholipase C
PMSG	Pregnant mare serum gonadotropin
RhoA	Ras homolog gene family, member A
SR1	5-(4-Chloro-phenyl)-1-(2,4-dichloro-phenyl)-4-methyl-1H-pyrazole-3-carboxylic acid piperidin-1-ylamide hydrochloride
SR2	5-(4-Chloro-3-methylphenyl)-1-(4-methylbenzyl)-*N*-((1S,2S,4R)-1,3,3-trimethylbicyclo[2.2.1]heptan-2-yl)-1H-pyrazole-3-carboxamide
TRPV1	Transient receptor potential vanilloid type 1 channel
ZF	Zona-free

## References

[1] Richards J.S., Russell D.L., Ochsner S., Hsieh M., Doyle K.H., Falender A.E., Sharma S.C. (2002). Novel signaling pathways that control ovarian follicular development, ovulation, and luteinization. Recent Prog. Horm. Res..

[2] Canipari R., Cellini V., Cecconi S. (2012). The ovary feels fine when paracrine and autocrine networks cooperate with gonadotropins in the regulation of folliculogenesis. Curr. Pharm. Des..

[3] Wang H., Dey S.K., Maccarrone M. (2006). Jekyll and hyde: Two faces of cannabinoid signaling in male and female fertility. Endocr. Rev..

[4] Brents L.K. (2016). Marijuana, the endocannabinoid system and the female reproductive system. Yale J. Biol. Med..

[5] Maccarrone M., Valensise H., Bari M., Lazzarin N., Romanini C., Finazzi-Agrò A. (2000). Relation between decreased anandamide hydrolase concentrations in human lymphocytes and miscarriage. Lancet.

[6] Maccarrone M., Fride E., Bisogno T., Bari M., Cascio M.G., Battista N., Di Marzo V. (2005). Up-regulation of the endocannabinoid system in the uterus of leptin knockout (ob/ob) mice and implications for fertility. Mol. Hum. Reprod..

[7] Park B., McPartland J.M., Glass M. (2004). Cannabis, cannabinoids and reproduction. Prostaglandins Leukot. Essent. Fatty Acids.

[8] Cecconi S., Rossi G., Castellucci A., D’Andrea G., Maccarrone M. (2014). Endocannabinoid signaling in mammalian ovary. Eur. J. Obstet. Gynecol. Reprod. Biol..

[9] Correa F., Wolfson M.L., Valchi P., Aisemberg J., Franchi A.M. (2016). Endocannabinoid system and pregnancy. Reproduction.

[10] Pertwee R.G., Pertwee R. (2015). Endocannabinoids and their pharmacological actions. Endocannabinoids. Handbook of Experimental Pharmacology.

[11] Maccarrone M. (2009). Endocannabinoids: Friends and foes of reproduction. Prog. Lipid Res..

[12] Maccarrone M., Bab I., Bíró T., Cabral G.A., Dey S.K., Di Marzo V., Konje J.C., Kunos G., Mechoulam R., Pacher P. (2015). Endocannabinoid signaling at the periphery: 50 years after THC. Trends Pharmacol. Sci..

[13] Rapino C., Battista N., Bari M., Maccarrone M. (2014). Endocannabinoids as biomarkers of human reproduction. Hum. Reprod. Update.

[14] Schuel H., Burkman L.J., Lippes J., Crickard K., Forester E., Piomelli D., Giuffrida A. (2002). N-Acylethanolamines in human reproductive fluids. Chem. Phys. Lipids.

[15] El-Talatini M.R., Taylor A.H., Konje J.C. (2009). Fluctuation in anandamide levels from ovulation to early pregnancy in in-vitro fertilization-embryo transfer women, and its hormonal regulation. Hum. Reprod..

[16] Ding J., Luo X.T., Yao Y.R., Xiao H.M., Guo M.Q. (2017). Investigation of changes in endocannabinoids and N-acylethanolamides in biofluids, and their correlations with female infertility. J. Chromatogr. A.

[17] Cui N., Yang Y., Xu Y., Zhang J., Jiang L., Hao G. (2017). Decreased expression of fatty acid amide hydrolase in women with polycystic ovary syndrome. Gynecol. Endocrinol..

[18] Almada M., Amaral C., Diniz-da-Costa M., Correia-da-Silva G., Teixeira N.A., Fonseca B.M. (2016). The endocannabinoid anandamide impairs in vitro decidualization of human cells. Reproduction.

[19] Bagavandoss P., Grimshaw S. (2010). Temporal and spatial distribution of the cannabinoid receptors (CB_1_, CB_2_) and fatty acid amide hydroxylase in the rat ovary. Anat. Rec. (Hoboken).

[20] El-Talatini M.R., Taylor A.H., Elson J.C., Brown L., Davidson A.C., Konje J.C. (2009). Localisation and function of the endocannabinoid system in the human ovary. PLoS ONE.

[21] Peralta L., Agirregoitia E., Mendoza R., Expósito A., Casis L., Matorras R., Agirregoitia N. (2011). Expression and localization of cannabinoid receptors in human immature oocytes and unfertilized metaphase-II oocytes. Reprod. Biomed. Online.

[22] Agirregoitia E., Ibarra-Lecue I., Totorikaguena L., Mendoza R., Expósito A., Matorras R., Agirregoitia N. (2015). Dynamics of expression and localization of the cannabinoid system in granulosa cells during oocyte nuclear maturation. Fertil. Steril..

[23] Agirregoitia E., Totorikaguena L., Expósito A., Mendoza R., Matorras R., Agirregoitia N. (2016). Dynamic of expression and localization of cannabinoid-degrading enzymes FAAH and MGLL in relation to CB_1_ during meiotic maturation of human oocytes. Cell Tissue Res..

[24] López-Cardona A.P., Sánchez-Calabuig M.J., Beltran-Breña P., Agirregoitia N., Rizos D., Agirregoitia E., Gutierrez-Adán A. (2016). Exocannabinoids effect on in vitro bovine oocyte maturation via activation of AKT and ERK1/2. Reproduction.

[25] López-Cardona A.P., Pérez-Cerezales S., Fernández-González R., Laguna-Barraza R., Pericuesta E., Agirregoitia N., Agirregoitia E. (2017). CB_1_ cannabinoid receptor drives oocyte maturation and embryo development via PI3K/Akt and MAPK pathways. FASEB J..

[26] Oddi S., Totaro A., Scipioni L., Dufrusine B., Stepniewski T.M., Selent J., Dainese E. (2018). Role of palmitoylation of cysteine 415 in functional coupling CB_1_ receptor to Gαi2 protein. Biotechnol. Appl. Biochem..

[27] Horner K., Livera G., Hinckley M., Trinh K., Storm D., Conti M. (2003). Rodent oocytes express an active adenylyl cyclase required for meiotic arrest. Dev. Biol..

[28] Vaccari S., Horner K., Mehlmann L.M., Conti M. (2008). Generation of mouse oocytes defective in cAMP synthesis and degradation: Endogenous cyclic AMP is essential for meiotic arrest. Dev. Biol..

[29] Susor A., Jansova D., Anger M., Kubelka M. (2016). Translation in the mammalian oocyte in space and time. Cell Tissue Res..

[30] Oddi S., Dainese E., Sandiford S., Fezza F., Lanuti M., Chiurchiù V., Maccarrone M. (2012). Effects of palmitoylation of Cys(415) in helix 8 of the CB(1) cannabinoid receptor on membrane localization and signalling. Br. J. Pharmacol..

[31] Hirohashi N., Yanagimachi R. (2018). Sperm acrosome reaction: Its site and role in fertilization. Biol. Reprod..

[32] Yang Z.M., Paria B.C., Dey S.K. (1996). Activation of brain-type cannabinoid receptors interferes with preimplantation mouse embryo development. Biol. Reprod..

[33] Paria B.C., Dey S.K. (2000). Ligand-receptor signaling with endocannabinoids in preimplantation embryo development and implantation. Chem. Phys. Lipids.

[34] Buckley N.E., Hansson S., Harta G., Mezey E. (1998). Expression of the CB_1_ and CB_2_ receptor messenger RNAs during embryonic development in the rat. Neuroscience.

[35] Conti M. (2011). Phosphodiesterases and regulation of female reproductive function. Curr. Opin. Pharmacol..

[36] Gilchrist R.B., Luciano A.M., Richani D., Zeng H.T., Wang X., Vos M.D., Thompson J.G. (2016). Oocyte maturation and quality: Role of cyclic nucleotides. Reproduction.

[37] Richani D., Gilchrist R.B. (2018). The epidermal growth factor network: Role in oocyte growth, maturation and developmental competence. Hum. Reprod. Update.

[38] Lowther K.M., Nikolaev V.O., Mehlmann L.M. (2011). Endocytosis in the mouse oocyte and its contribution to cAMP signaling during meiotic arrest. Reproduction.

[39] Nader N., Dib M., Daalis A., Kulkarni R.P., Machaca K. (2014). Role for endocytosis of a constitutively active GPCR (GPR185) in releasing vertebrate oocyte meiotic arrest. Dev. Biol..

[40] den Boon F.S., Chameau P., Schaafsma-Zhao Q., van Aken W., Bari M., Oddi S., Werkman T.R. (2012). Excitability of prefrontal cortical pyramidal neurons is modulated by activation of intracellular type-2 cannabinoid receptors. Proc. Natl. Acad. Sci. USA.

[41] Tiwari M., Prasad S., Shrivastav T.G., Chaube S.K. (2017). Calcium signaling during meiotic cell cycle regulation and apoptosis in mammalian oocytes. J. Cell Physiol..

[42] Willoughby D., Cooper D.M.F. (2007). Organization and Ca^2+^ regulation of adenylyl cyclases in cAMP microdomains. Physiol. Rev..

[43] Thomas P. (2017). Role of G-protein-coupled estrogen receptor (GPER/GPR30) in maintenance of meiotic arrest in fish oocytes. J. Steroid Biochem. Mol. Biol..

[44] Bagavandoss P., Grimshaw S. (2012). Distribution of adenylyl cyclases in the rat ovary by immunofluorescence microscopy. Anat. Rec. (Hoboken).

[45] Gaetz J., Kapoor T.M. (2004). Dynein/dynactin regulate metaphase spindle length by targeting depolymerizing activities to spindle poles. J. Cell Biol..

[46] Bennabi I., Terret M.E., Verlhac M.H. (2016). Meiotic spindle assembly and chromosome segregation in oocytes. J. Cell Biol..

[47] Chen F., Jiao X.F., Zhang J.Y., Wu D., Ding Z.M., Wang Y.S., Huo L.J. (2018). Nucleoporin35 is a novel microtubule associated protein functioning in oocyte meiotic spindle architecture. Exp. Cell Res..

[48] Tomari H., Honjo K., Kunitake K., Aramaki N., Kuhara S., Hidaka N., Horiuchi T. (2018). Meiotic spindle size is a strong indicator of human oocyte quality. Reprod. Med. Biol..

[49] Wang Z.W., Zhang G.L., Schatten H., Carroll J., Sun Q.Y. (2016). Cytoplasmic determination of meiotic spindle size revealed by a unique inter-species germinal vesicle transfer model. Sci. Rep..

[50] Radford S.J., Nguyen A.L., Schindler K., McKim K.S. (2017). The chromosomal basis of meiotic acentrosomal spindle assembly and function in oocytes. Chromosoma.

[51] Lauckner J.E., Jensen J.B., Chen H.Y., Lu H.C., Hille B., Mackie K. (2008). GPR55 is a cannabinoid receptor that increases intracellular calcium and inhibits M current. Proc. Natl. Acad. Sci. USA.

[52] Schuel H., Burkman L.J., Lippes J., Crickard K., Mahony M.C., Giuffrida A., Makriyannis A. (2002). Evidence that anandamide-signaling regulates human sperm functions required for fertilization. Mol. Reprod. Dev..

[53] Amoako A.A., Marczylo T.H., Elson J., Taylor A.H., Willets J.M., Konje J.C. (2014). Relationship between seminal plasma levels of anandamide congeners palmitoylethanolamide and oleoylethanolamide and semen quality. Fertil. Steril..

[54] Cecconi S., Rossi G., Santilli A., Stefano L.D., Hoshino Y., Sato E., Macchiarelli G. (2010). Akt expression in mouse oocytes matured in vivo and in vitro. Reprod. Biomed. Online.

[55] Compagnucci C., Di Siena S., Bustamante M.B., Di Giacomo D., Di Tommaso M., Maccarrone M., Sette C. (2013). Type-1 (CB_1_) cannabinoid receptor promotes neuronal differentiation and maturation of neural stem cells. PLoS ONE.

[56] Pucci M., D’Addario C. (2016). Assessing Gene Expression of the Endocannabinoid System. Methods Mol. Biol..

[57] Pucci M., Pasquariello N., Battista N., Di Tommaso M., Rapino C., Fezza F., Maccarrone M. (2012). Endocannabinoids stimulate human melanogenesis via type-1 cannabinoid receptor. J. Biol. Chem..

[58] Rossi G., Palmerini M.G., Macchiarelli G., Buccione R., Cecconi S. (2006). Mancozeb adversely affects meiotic spindle organization and fertilization in mouse oocytes. Reprod. Toxicol..

[59] Di Nisio V., Rossi G., Palmerini M.G., Macchiarelli G., Tiboni G.M., Cecconi S. (2018). Increased rounds of gonadotropin stimulation have side effects on mouse fallopian tubes and oocytes. Reproduction.

[60] Coticchio G., Rossi G., Borini A., Grøndahl C., Macchiarelli G., Flamigni C., Cecconi S. (2004). Mouse oocyte meiotic resumption and polar body extrusion in vitro are differentially influenced by FSH, epidermal growth factor and meiosis-activating sterol. Hum. Reprod..

[61] Sanfins A., Lee G.Y., Plancha C.E., Overstrom E.W., Albertini D.F. (2003). Distinctions in meiotic spindle structure [1] and assembly during in vitro and in vivo maturation of mouse oocytes. Biol. Reprod..

